# Coexpression within Integrated Mitochondrial Pathways Reveals Different Networks in Normal and Chemically Treated Transcriptomes

**DOI:** 10.1155/2014/452891

**Published:** 2014-06-24

**Authors:** Cong Chen, Tae Kyung Hyun, Xiao Han, Zhihui Feng, Yuan Li, Xiaolong Liu, Jiankang Liu

**Affiliations:** ^1^Center for Mitochondrial Biology and Medicine, The Key Laboratory of Biomedical Information Engineering of Ministry of Education, School of Life Science and Technology and Frontier Institute of Science and Technology, Xi'an Jiaotong University, Xi'an 710049, China; ^2^Division of Applied Life Science (Brain Korea 21-World Class University Program), Plant Molecular Biology and Biotechnology Research Center, Gyeongsang National University, Jinju 660-701, Republic of Korea; ^3^The Liver Center of Fujian Province, Fujian Medical University, Fuzhou 350025, China

## Abstract

As energy producers, mitochondria play a pivotal role in multiple cellular processes. Although several lines of evidence suggest that differential expression of mitochondrial respiratory complexes (MRCs) has a significant impact on mitochondrial function, the role of integrated MRCs in the whole coexpression network has yet to be revealed. In this study, we construct coexpression networks based on microarray datasets from different tissues and chemical treatments to explore the role of integrated MRCs in the coexpression network and the effects of different chemicals on the mitochondrial network. By grouping MRCs as one seed target, the hypergeometric distribution allowed us to identify genes that are significantly coexpress with whole MRCs. Coexpression among 46 MRC genes (approximately 78% of MRC genes tested) was significant in the normal tissue transcriptome dataset. These MRC genes are coexpressed with genes involved in the categories “muscle system process,” “metabolic process,” and “neurodegenerative disease pathways,” whereas, in the chemically treated tissues, coexpression of these genes mostly disappeared. These results indicate that chemical stimuli alter the normal coexpression network of MRC genes. Taken together, the datasets obtained from the different coexpression networks are informative about mitochondrial biogenesis and should contribute to understanding the side effects of drugs on mitochondrial function.

## 1. Introduction

Mitochondria are small membrane-enclosed organelles (from 0.5 to 1.0 *μ*M in diameter) found in most eukaryotic cells except mature red blood corpuscles [1]. Mitochondria are the powerhouses of eukaryotic cells and are involved in many cellular processes, including apoptosis; ion homeostasis; and the metabolism of glucose, lipids, and amino acids [2]. ATP, the energy currency of cell, is the final product of the respiratory chain/oxidative phosphorylation system, which consists of five protein complexes (complexes I–V) localized to the inner mitochondrial membrane [3]. Mitochondrial defects including mitochondrial DNA (mtDNA) mutations, altered expression and activity of respiratory chain subunits and glycolytic enzymes, and decreased oxidation of NADH-linked substrates have been suspected to play an important role in the development and progression of diseases, such as certain neurodegenerative diseases, diabetes, Leigh's disease, and cancer [4–7]. These clinical phenotypes are direct evidence reflecting the essential function of mitochondria. Mitochondrial genetic disorders are caused by defects in nuclear or mtDNA that affect the expression of the mtDNA-encoded mitochondrial respiratory complexes (MRCs) and the biosynthesis of the mtDNA-encoded polypeptides [8]. Mutations in genes required for mtDNA maintenance, expression, and replication regulate genetic disorders, indicating that differential expression of MRCs and related genes has a significant impact on mitochondrial dysfunction [7, 9]. Therefore, systematic analysis of nuclear and mitochondrial gene expression in the context of well-defined disease models should provide insight into the interaction of gene regulatory networks with MRCs, improving our understating of mitochondrial disorders.

Coexpression analysis using transcriptome datasets generated by high-throughput microarray transcript profiling produces correlations that have often been considered to imply functional relationships [10, 11]. A strong correlation among transcripts for MRC components has been found by this type of coexpression analysis in plants. In the case of plant MRC genes, it has been shown that genes belonging to MRCs are clustered into the same coexpression group [10]. Similarly, several mtDNA-encoded mitochondrial genes form a small cluster with a nuclear-encoded mitochondrial gene module and the glycolysis module [12]. Coexpression analysis has also implicated several unannotated genes in cancer and mitochondrial complex I disease [13], indicating that coexpression analysis is a useful tool not only for understanding many diseases at the molecular level but also for identification of novel candidate genes involved in mitochondria-related diseases. Although several studies have demonstrated the power of coexpression analysis, few have exploited MRCs as an integrated component for analyzing the coexpression network.

To further investigate the role of integrated MRCs in the whole coexpression network, we determined the coexpression networks of normal and chemically treated human tissues by analysis of Pearson correlation factors. In the coexpression network under normal conditions, we found that the MRCs are almost fully self-connected. This self-connection indicates that whole MRCs might play a role similar to that of single genes in the coexpression network. Using the hypergeometric distribution, we considered genes with a *P* value less than 10^−8^ to be coexpression “friends” with MRCs. Candidate functions for these “friends” were determined through enrichment analysis, using Gene Ontology (GO) terms and Kyoto Encyclopedia of Genes and Genomes (KEGG) pathways. We then explored the coexpression network between “friends” and MRCs in both normal and chemically treated tissues. The systematic coexpression network of genes interacting with whole MRCs identifies candidates that potentially participate in mitochondrial biogenesis and could serve as targets for future therapeutic interventions aimed at modulating mitochondrial function.

## 2. Methods

### 2.1. Selection of Datasets and Construction of Coexpression Networks

To create coexpression “friends” with MRCs, we first constructed a genome-wide coexpression networks using two different microarray datasets. Expression datasets for 65 human tissues were downloaded from the COXPRESdb website (http://coxpresdb.jp/). Transcription profiles (E-MTAB-798) of human hepatocytes treated with 130 chemical compounds including drugs such as acetaminophen, aspirin, rifampicin, metformin hydrochloride, simvastatin, and tamoxifen citrate were obtained from EMBL-EBI (http://www.ebi.ac.uk/arrayexpress/). Coexpression networks were constructed as described in Azuaje [14]. The Spearman coexpression coefficient, *ρ*, was calculated for each pair of genes, and all gene pairs with *ρ* ≥ 0.3 were defined as gene-gene associations in the network. In the coexpression network, the nodes represent genes and the edges represent the connection with coefficient ≥0.3.

### 2.2. Analysis of Coexpression Significance

The MRC is designated as NADH-Coenzyme Q reductase (complex I), succinate-CoQ reductase (complex II), ubiquinol-cytochrome c reductase (complex III), cytochrome c oxidase (complex IV), and ATP synthase (complex V). Complexes I, II, III, and IV play as the electron transfer complexes, whereas complex V is known as an enzyme-conserving complex [3]. Since different datasets contain different probes mapping to different gene symbols, we used gene symbols that are present in gene platform file containing 56 MRC genes (33 genes for complex I, 4 genes for complex II, 8 genes for complex III, and 23 genes for complex IV). To determine coexpression significance, 56 MRC genes were selected as an integrated target seed for further analysis. The hypergeometric distribution was used to calculate the connection between the MRC genes and other genes in the whole coexpression network. This discrete probability distribution describes the probability of *k* successes in *n* draws, without replacement, from a finite population of size *N* containing *K* samples. For example, suppose that there are *K* (56) MRC genes among *N* (20,000) genes in the genome. Gene X makes connections with *k* MRCs and *n* connections with the genome. We define genes with a *P* value less than 10^−8^ to be coexpression “friends” with MRCs.

### 2.3. Software Tools

The R platform (http://www.r-project.org/) was used for network generation and statistical calculations. Functional annotation of “friends” was carried out using the web based tool PANTHER (http://www.pantherdb.org/). GO enrichment and KEGG analysis were performed with DAVID (http://david.abcc.ncifcrf.gov/). DAVID was also used to analyze the functional annotations of the gene sets and modules. For pathway enrichment analysis of the MCR coexpressed genes, “GO_BP,” “KEGG_PATHWAY,” and “PANTHER_PATHWAY” were selected. The *P* values and a modified Fisher's exact test were used to determine the enrichment of gene sets in ontology.

## 3. Results and Discussion

### 3.1. Coexpression of MRC Genes across Different Human Tissues

**Figure 1:**
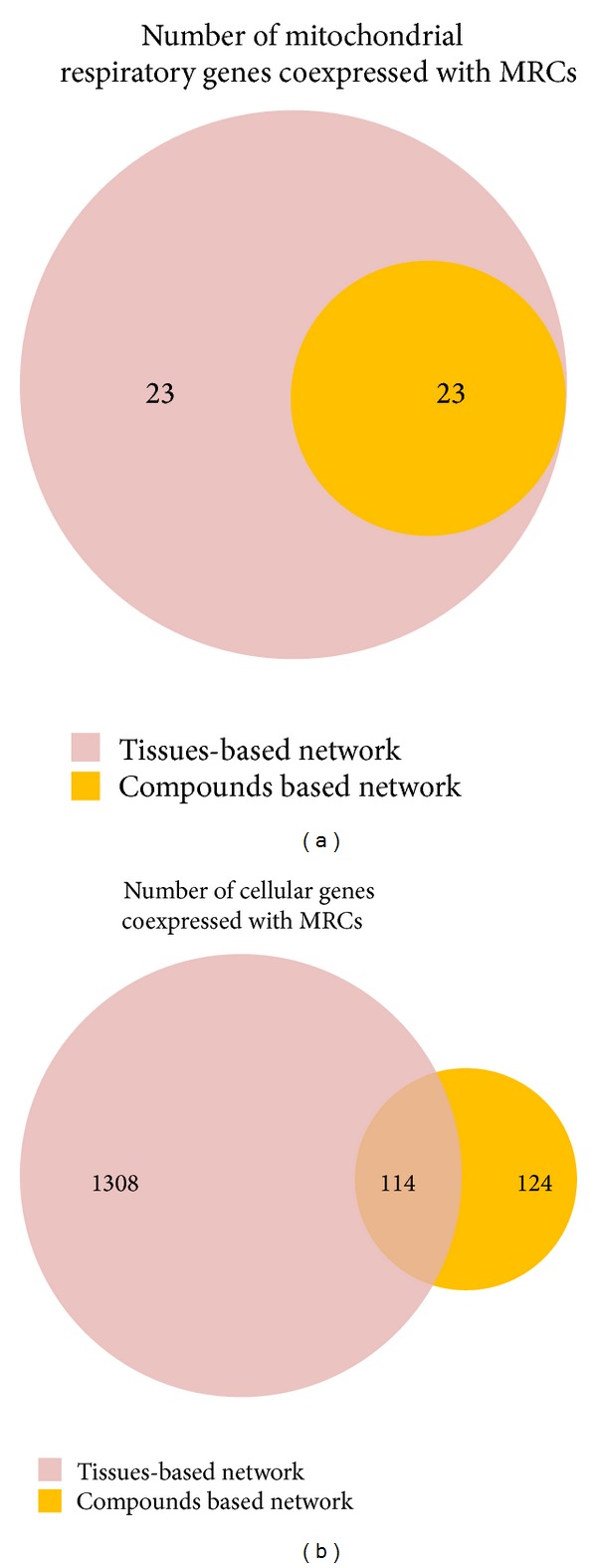
Genes significantly coexpressed with MRCs in normal and chemically treated tissues. (a) Self-connections among mitochondrial respiratory complexes. (b) Coexpressed cellular genes.

**Figure 2:**
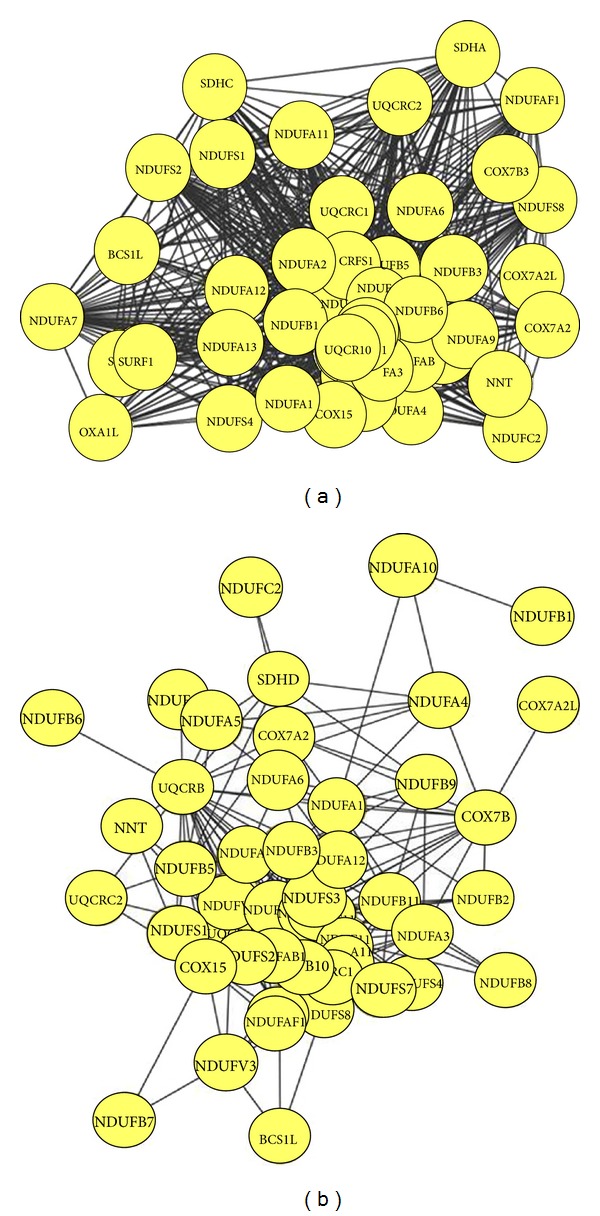
Genes coexpressed with MRCs in (a) the tissue-based network and (b) the chemical treatment-based network.

Two functional entities are involved in the generation of ATP by a process called oxidative phosphorylation located in the mitochondrial inner membrane [3]. The first entity is the electron transfer chain historically defined as four complexes (I, II, III, and IV), whereas the second entity is known as the system that phosphorylates ADP to produce ATP [15]. Among these complexes, complex I is the first and largest enzyme complex of the respiratory chain and is directly involved in maintaining cellular reduction-oxidation (redox; NADH/NAD^+^) homeostasis [15]. The mammalian complex I is composed of at least 45 subunits and is the main source of reactive oxygen species, which are implicated in cell signaling, disease, and aging [16]. Its deficiency is the most frequently encountered in mitochondrial disorders [17], and the large number of genes coding for complex I subunits might explain why complex I deficiency is characterized by marked clinical and genetic heterogeneity [18]. Complex II is composed of four nuclear-encoded subunits, whereas complex III is a complex of 11 subunits [19, 20]. Complex II receives electrons via FADH2 and transfers it to complex III thought Coenzyme Q 10. Then electrons are carried by cytochrome c to complex IV, which is composed of 19 subunits. This electron transport is required for the generation of the transmembrane proton gradient in inner mitochondrial membrane which is utilized by complex V to convert ADP to ATP [21]. Defect in any of MRCs leads to impaired ATP production and results in a mitochondrial disease involving abnormality of the central nervous system and eyes, renal, muscle, heat, and haematological system, as well as diverse age-related disorders including cancer and degenerative diseases [21–24]. This indicates that these complexes have a significant impact on mitochondrial function. To investigate the connectivity of expression between MRCs and other genes, we generated coexpression networks using expression datasets for 65 human tissues. Prior genome-wide expression analyses have demonstrated significant coexpression of MRC genes under various physiological conditions in several species [25]. Similarly, our large-scale analysis across different human tissues reveals a coexpression cluster (46 out of 56 MRC genes) significantly enriched in genes belonging to mitochondrial complexes I to IV (Figure 1(a)). Of these, 37 genes belong to complex I, composed of 45 subunits [26]. Mitochondrial complex I uses NADH as a cofactor for electron transfer and translocates protons across the inner mitochondrial membrane [27]. The genes NDUFB5 (*P* = 9.98*E* − 55) and NDUFA7 (*P* = 1.14*E* − 54), two subunits of complex I, exhibited the lowest *P* values (Supplementary Table S1 available online at http://dx.doi.org/10.1155/2014/452891), indicating that these genes are significantly coexpressed with other MRCs. In many organisms, the complexes I, III, and IV can associate into supercomplexes [28–30]. Among the various types of association, the I + III_2_ + IV_1–4_ supercomplex or the respirasome is one of the most intriguing supercomplexes, because it considered the minimal unit to perform complete respiration from NADH to oxygen [29, 31]. This supercomplex has also been detected by inhibitor titration in bovine mitochondria, suggesting that the two mitochondrial electron transfer complexes specifically interact to form this supercomplex. In our coexpression network (Figure 2(a)), NDUFB5 is coexpressed with SDHC of complex II and UQCRC2 of complex III. In addition, UQCRC1 in mitochondrial complex III is significantly coexpressed with the complex I subunits NDUFA13, NDUFAF1, and NDUFS7. It has been shown that the absence in complex III results in a dramatic loss of complex I in humans, and complex I is necessary for fully assembled complex III [32–34], indicating that supercomplex formation is necessary for assembly and stability of individual components [35]. Taken together, these results suggest that complex I is tightly coexpressed with complex III compared to other complexes and that this coexpression might be required for maintaining the supercomplex.

### 3.2. Coexpression of MRC Genes in Chemically Treated Tissues

Drug-induced liver injury is a common side effect of certain pharmaceutical therapies. Drugs can be metabolized into electrophilic chemicals or free radicals, which have direct effects upon mitochondrial proteins. Damage to mitochondrial proteins decreases their affinity for substrates, resulting in mitochondrial dysfunction [36, 37]. Therefore, understanding drug-induced mitochondrial toxicity is critical for the development of safe drugs. To investigate the effect of chemical toxicity on the coexpression network of MRC genes in liver, transcription profiles of human hepatocytes treated with 130 chemical compounds were used as input. Stress-related stimuli induce the remodeling of coexpression networks, resulting in the large-scale alteration of cellular function, involving a shift of resources from growth and metabolism to protection and maintenance [38, 39]. As shown in Figure 1(b), coexpression of MRC genes in treated human hepatocytes was significantly lower than in nontreated human tissues. NDUFAF4, a subunit of complex I, exhibited significant coexpression with other MRCs under normal conditions (*P* = 2.31*E* − 54; Supplementary Table S1). However, the *P* value between NDUFAF4 and other MRCs increased substantially (*P* = 0.209) after chemical treatment, indicating a major change in the coexpression network. UQCRC2, a complex III subunit, is also tightly connected with other MRCs in normal tissue (*P* = 1.16*E* − 52). Again, this connection disappeared under chemical treatment (*P* = 0.001). A similar loss of coexpression was observed for 23 MRC genes, although the remaining 23 genes (19 from complex I, 2 from complex III, and 2 from complex IV) were still highly coexpressed (Figure 2(b)). NDUFA1 is coexpressed with complex I subunit NDUFA4 and complex III subunit UQCRB. One possible explanation for these changes in coexpression in response to chemical treatment is that these compounds directly or indirectly influence MRC gene expression. Indeed, differential expression of mitochondrial genes has been induced by manipulating the agonal-pH state and through drug treatment [40, 41]. Furthermore, some compounds might modulate cellular redox levels or dissipate the mitochondrial membrane gradient by facilitating anion flux across the mitochondrial inner membrane, as suggested by Toogood [42], resulting in remodeling of the coexpression network.

### 3.3. Coexpression of MRC and Cellular Genes

**Figure 3:**
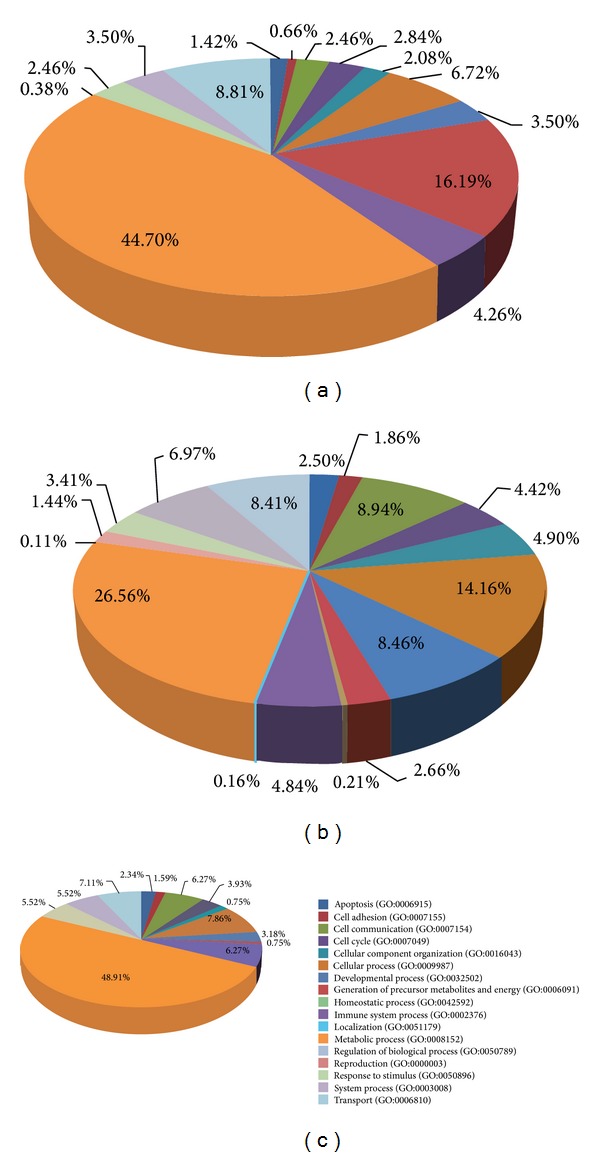
PANTHER analysis of the functional categories of genes coexpressed with MRCs. (a) Genes coexpressed in both normal tissues and chemically treated tissues. (b) Genes coexpressed with MRCs only in the tissue-based network. (c) Genes coexpressed with MRCs only in the chemically treated tissue.

In normal tissue, 1,422 genes are coexpressed significantly with MRC genes. Of these, coexpression of 1,308 genes was observed in normal tissue but not in chemically treated tissue. The remaining 114 genes are coexpressed in both normal and treated tissues. To identify the function of these 114 genes, we analyzed their associated GO terms using the online PANTHER tool (http://www.pantherdb.org/geneListAnalysis.do). A total of 51 (44.7%) genes were assigned to “metabolic process” (Figure 3(a)), indicating that this process is closely related to mitochondrial function. “Immune system process” and “apoptosis” were represented by 4.26% and 1.42% of these genes, respectively. The 1,308 genes that are coexpressed only in normal tissues were also categorized using PANTHER (Figure 3(b)). Of these, 26.56% were assigned to “metabolic processes” and 14.16% to “cellular processes.” “Immune system process” and “apoptosis” were assigned to 4.84% and 2.50% of these genes, respectively. AIFM1 (apoptosis-inducing factor, mitochondrion-associated 1) ENDOG (endonuclease G) is involved in intrinsic (mitochondria-associated) pathway for cancer cell apoptosis, for example, tightly coexpressed with MRC genes (*P* = 9.09*E* − 29 and 1.71*E*−39, resp.) in normal tissue, but this coexpression disappeared in treated tissues (Supplementary Table S1). AIFM1 is known to be important for the assembly and stability of complexes I and III [43]. In addition, the mutation or inhibition of MRC is widespread in cancer and intimately connected to apoptosis resistance [44], indicating that the MRC plays as a modulator of apoptosis for the treatment of cancer [45, 46]. Coexpression among MRC genes and the mechanistic target of rapamycin (mTOR) gene also disappeared after chemical treatment (*P* = 0.99; Supplementary Table S1). Disruption of the mTOR complex by treatment with the mTOR inhibitor rapamycin reduces mitochondrial membrane potential, oxygen consumption, and ATP synthetic capacity, indicating that formation of the mTOR complex is required for overall mitochondrial activity [47]. Taken together, these findings indicate that coexpression of MRC genes with cellular genes such as AIFM1 might be required to maintain the mitochondrial complexes. Furthermore, the disruption of this coexpression by chemical treatment suggests that similar disruptions might be responsible for mitochondria-related side effects of pharmaceuticals.

In contrast to the coexpression network in normal tissues, only 238 genes were significantly enriched in chemically treated tissues. Of these, 114 are coexpressed in normal tissues, whereas 124 are coexpressed only in treated tissues. This finding suggests that chemical treatment alters the coexpression network between MRC genes and cellular genes. Of the genes coexpressed only in treated tissues, almost half (48.91%) were associated with the term “metabolic process” (Figure 3(c)). The gene for 2,4-dienoyl CoA reductase 2 (DECR2), an auxiliary enzyme in the mitochondrial beta-oxidation of unsaturated fatty acids, is coexpressed with a *P* value of 7.49*E* − 8 (Supplementary Table S1). Three acyl-CoA thioesterases (ACOTs), ACOT11 (*P* = 5.51*E* − 42), ACOT13 (*P* = 1.14*E* − 26), and ACOT2 (*P* = 1.83*E* − 14), involved in peroxisomal lipid metabolism [48], were highly coexpressed with MRCs in normal tissue, whereas ACOT8 (*P* = 1.75*E* − 08) is coexpressed with MRCs in treated tissues (Supplementary Table S1).

Coexpression network, which is the reconstruction of biological networks from high-throughput data, can be used to identify higher-level features of gene-gene relationships based on graph theoretic considerations such as clustering coefficient or node degree [49, 50]. However, large-scale analyses only provide clues that help in forming a hypothesis [51]. Although the differences among coexpression networks (Supplementary Table S1) should help identify and prioritize candidate genes to determine the effects of drugs on mitochondria, further study is required to determine the relationship between the coexpressed genes and specific mitochondrial functions.

### 3.4. Functional Enrichment Analysis of MRC “Friends”

To investigate the biological processes represented by genes significantly coexpressed with MRC genes in normal tissue compared to treated tissues, we performed GO term enrichment analyses using the functional annotation tool DAVID (http://david.abcc.ncifcrf.gov/). For normal tissue, annotations for 1,308 genes were enriched in 17 terms, including “muscle system process,” “cellular metabolic process,” and “carboxylic acid metabolic process” (Table 1). For chemically treated tissues, GO term enrichment of 124 genes coexpressed with MRC genes found only ribosome biogenesis terms, such as “translational elongation and translation” (Table 2). A sufficient supply of ATP is required to maintain the contractile function of muscle [52], suggesting the importance of mitochondria during muscle contraction. Myosin provides energy and plays a vital role in muscle contraction. Myosin genes could be divided into several classifications [53], such as myosin heavy chain 1 (MYH1), myosin light chain 3 (MYL3), and myosin binding protein C2 (MYBPC2). Mutations in myosin genes lead to hypertrophic cardiomyopathy [54]. These findings indicate that muscle system closely interacts with MRC genes for improving mitochondrial function.

For the GO term “fatty acid metabolic process,” 39 genes were identified as “friends” of MRCs. Of these, carnitine palmitoyltransferase 2 (CPT2) localizes to the inner leaflet of the inner mitochondrial membrane, where it oxidizes long-chain fatty acids to produce substrates for the mitochondrial fatty acid beta-oxidation pathway [55]. Most of the genes required for mitochondrial biogenesis are controlled by DNA-binding transcription factors and coregulators [56]. Peroxisome proliferator-activated receptor gamma coactivator 1-alpha (PPARGC1A) is a transcriptional coactivator that regulates various metabolic processes including mitochondrial biogenesis and respiration [57]. Therefore, coexpression of MRC genes with functional genes such as CPT2 and PPARGC1A might be required for the function and maintenance of mitochondria.

### 3.5. Coexpression of MRC Genes in Neurodegenerative Disease Pathways

Several molecular, cellular, biochemical, and animal model studies have suggested that mitochondrial dysfunction closely relates to the progression of several neurodegenerative diseases [58]. Using KEGG pathway enrichment analysis, we found that 19 MRC genes (41% of MRC genes in the coexpression network), belonging to complexes I to IV, are coregulated with Parkinson's, Alzheimer's, and Huntington's disease pathways (Table 3), indicating the importance of MRCs in neurodegenerative disease pathways. In Parkinson's disease, PTEN-induced putative kinase 1 (PINK1), a mitochondrial serine/threonine-protein kinase, was found to be a coexpression “friend” with MRC genes in normal tissue (*P* = 1.28*E* − 13) but not in treated tissues (*P* = 0.35) (Supplementary Table S1). PINK1 loss-of-function causes mitochondrial dysfunction and Parkinsonism [59]. In addition, the *α*1 subunit of dihydropyridine receptor (CACNA1S), calmodulin-like 6 (CALML6), presenilin protein 2 (PSEN2), BCL2-associated agonist of cell death (BAD), and lipoprotein lipase (LPL) in Alzheimer's disease pathway are significantly coexpressed with MRC genes (Table 3), whereas clathrin light chain B (CLTB), clathrin heavy polypeptide-like 1 (CLTCL1), DNA-directed RNA polymerase II subunits (POLR2E, POLR2L), and PPARGC1A were identified as coexpression “friends” with MRC genes in Huntington's disease pathway. These findings indicate that MRC genes are directly or indirectly linked with neurodegenerative disease pathways. Mitochondrial dysfunction causes not only neurodegenerative diseases but also hypertrophic and dilated cardiomyopathy [60]. MRC “friend” genes were enriched in the hypertrophic and dilated cardiomyopathy pathways in normal tissue (Table 3), whereas coexpression again disappeared after treatment with chemicals. KEGG pathway enrichment analysis thus suggests that the coexpression network revealed by using MRC genes as seed genes provides a possible link between mitochondria and various disease pathways.

## 4. Conclusion

In this study, we have compared the networks of genes coexpressed with MRC genes in normal and chemically treated tissues. We find a differential distribution of coexpression after chemical treatment. These differences might be mediated by chemical-related stimuli, suggesting that coexpression network analysis can provide helpful information for understanding side effects of drugs on mitochondrial functions.

## Supplementary Material

We added “Gene list of co-expressed with MRCs” in the Supplementary Material.

## Figures and Tables

**Table 1:** GO enrichment of genes coexpressed with MRCs only in normal tissue transcriptome.

Term	Count	Pop	*P* value	Genes
Muscle system process	68	168	1.15*E* − 35	*MYBPC2, TNNC2, MYBPC1, TNNC1, GNA11, MYBPC3, ANKRD2, PGAM2, KCNJ12, TTN, DES, DYSF, CHRNA1, MAP2K6, MB, ACTA1, * ***MYH1*** *, CRYAB, MYH2, TBCE, MYH4, * *MYLK2, ACTN2, PDE4D, MYH7, MYH6, CACNG1, MYH8, * *TNNT2, TRDN, TNNT3, TNNT1, PSEN2, RYR1, SMPX, RYR2, * *KBTBD10, STBD1, CASQ2, SGCA, CLCN1, ALDOA, MYL7, * *MYL4, TCAP, MYL3, MYL1, DAG1, MYOT, MYOM2, CKMT2, * *CAMK2D, MYOM1, SCN5A, HRC, ACTC1, MSTN, TRIM63, * *TNNI3, HOMER1, CACNA1S, TNNI2, SLC6A8, CHRNB4, * *GAMT, CHRNB1, SCN4A, SNTA1 *
Cellular ketone metabolic process	106	567	7.46*E* − 23	
Carboxylic acid metabolic process	99	556	8.48*E* − 20	
Oxoacid metabolic process	99	556	8.48*E* − 20	
Glucose metabolic process	41	153	2.39*E* − 14	*PRKAG3, ALDOA, LDHA, PHKA1, PRKAG2, PGAM2, OGDH, PDHB, ACN9, HIBADH, PPP1R3C, PPP1R1A, GYS1, ENO3, PDHA1, GAPDH, AGL, PDK2, WDTC1, CRYAB, * *PHKG1, PDK4, EPM2A, BAD, DLAT, PPP1R3A, PFKM, * *FBP2, PPARGC1A, PPP1CB, GPI, PPP1R2, GBE1, PYGM, * *PGM1, GPT, PGK1, DCXR, UGP2, MDH2, MDH1 *
Hexose metabolic process	43	192	3.64*E* − 12	
Acetyl-CoA metabolic process	17	31	1.39*E* − 11	
Fatty acid metabolic process	39	198	2.11*E* − 09	*PRKAG3, ACOX1, PPARA, TYRP1, ACADSB, CYP2J2, PTGES2, ECH1, * ***CPT2*** *, PRKAG1, PRKAG2, ACOT2, * *NDUFAB1, ECHS1, HADHA, HADHB, PEX7, ACSL1, * *ACOT11, ETFDH, GNPAT, PRKAA2, HADH, CPT1B, LPL, * *PLA2G15, ACADM, ACADS, MCAT, PRKAB2, CRAT, * ***PPARGC1A*** *, C9ORF3, ACADVL, UCP3, ANKRD23, MLYCD, * *FABP3, MECR *

**Table 2:** GO enrichment of genes coexpressed with MRCs only in chemically treated tissue.

Term	Count	Pop hits	*P* value	Genes
Translational elongation	12	101	7.51*E* − 11	*RPS19, RPL32, RPL14, RPS29, RPL22, FAU, RPS10, RPL38, RPL12, RPS21, UBA52, RPL29 *
Translation	16	331	5.86*E* − 09	*RPL14, MRPS21, RPL38, RPL29, MRPL11, RPS19, RPL32, RPS29, EIF3H, RPL22, FAU, RPS10, MRPL48, RPL12, RPS21, UBA52 *
Metabolic process	73	7647	1.98*E* − 05	
Protein metabolic process	36	2812	1.18*E* − 04	
Primary metabolic process	66	6923	1.60*E* − 04	
Macromolecule metabolic process	56	5710	6.11*E* − 04	

**Table 3:** KEGG enrichment analysis of genes coexpressed with MRCs.

Term	Count	Pop	*P* value	Genes
Parkinson's disease	40	128	9.27*E* − 15	*UQCRC2, ATP5D, NDUFB4, NDUFB6, NDUFB7, UBE2G1, CYC1, NDUFAB1, * ***PINK1*** *, ATP5G2, UQCRFS1, COX5A, NDUFB1, NDUFB2, UQCR11, ATP5O, ATP5H, NDUFS1, ATP6, NDUFA5, NDUFA2, COX7A1, SLC25A4, NDUFA6, CYCS, NDUFC2, ATP5F1, COX4I1, NDUFC1, NDUFA10, VDAC2, VDAC3, VDAC1, SDHA, NDUFV3, PPID, NDUFV2, SDHD, COX6A2, ATP5A1 *
Hypertrophic cardiomyopathy (HCM)	32	85	2.33*E* − 14	*PRKAG3, MYL2, TNNC1, MYL3, PRKAG1, MYBPC3, PRKAG2, CACNB1, DAG1, TPM2, TTN, TPM3, DES, DMD, ITGB6, PRKAA2, ACTC1, CACNG6, PRKAB2, MYH7, MYH6, CACNG1, TNNI3, * ***CACNA1S*** *, TNNT2, ATP2A2, SGCG, ITGA7, SGCD, RYR2, SGCA, SGCB *
Alzheimer's disease	41	163	1.03*E* − 11	*UQCRC2, ATP5D, NDUFB4, NDUFB6, NDUFB7, CYC1, NDUFAB1, ATP5G2, UQCRFS1, COX5A, NDUFB1, NDUFB2, UQCR11, * ***CALML6*** *, ATP5O, ATP5H, GAPDH, NDUFS1, ATP6, NDUFA5, * ***LPL*** *, NDUFA2, COX7A1, NDUFA6, CYCS, NDUFC2, ATP5F1, COX4I1, * ***BAD*** *, NDUFC1, NDUFA10, CACNA1S, SDHA, NDUFV3, ATP2A2, ATP2A1, NDUFV2, SDHD, * ***PSEN2*** *, COX6A2, ATP5A1 *
Dilated cardiomyopathy	30	92	1.15*E* − 11	*MYL2, TNNC1, MYL3, MYBPC3, CACNB1, DAG1, TTN, TPM2, TPM3, DES, DMD, ITGB6, PRKACA, ACTC1, CACNG6, MYH7, MYH6, CACNG1, TNNI3, CACNA1S, TNNT2, ADCY9, ATP2A2, SGCG, PLN, ITGA7, SGCD, RYR2, SGCA, SGCB *
Huntington's disease	43	180	1.80*E* − 11	*UQCRC2, ATP5D, NDUFB4, * ***POLR2E*** *, NDUFB6, * ***CLTB*** *, NDUFB7, * ***POLR2LL*** *, CYC1, NDUFAB1, ATP5G2, UQCRFS1, COX5A, NDUFB1, NDUFB2, UQCR11, ATP5O, ATP5H, NDUFS1, ATP6, NDUFA5, NDUFA2, COX7A1, SLC25A4, NDUFA6, CYCS, ATP5F1, NDUFC2, COX4I1, NDUFC1, NDUFA10, VDAC2, VDAC3, * ***PPARGC1A*** *, VDAC1, SDHA, NDUFV3, PPID, NDUFV2, SDHD, COX6A2, ATP5A1, * ***CLTCL1***
